# Supplementary material for: A tripartite microbial co-culture system for de novo biosynthesis of diverse plant phenylpropanoids
Source: Nat Commun. 2023 Jul 24;14:4448. doi: 10.1038/s41467-023-40242-9 (PMC10366228; doi:10.1038/s41467-023-40242-9)
Supplement: Supplementary file 1 — Supplementary Information [file 41467_2023_40242_MOESM1_ESM.pdf]

Supplementary Information for

A tripartite microbial co-culture system for de novo biosynthesis of diverse plant phenylpropanoids

Brooks *et al.*

Supplementary Table 1: Plasmids and primers used in this study

Plasmid Name	Cloning Method	Primers 5'->3'(Binding sequence in lowercase, overhang in uppercase)	Source
pRSFDuet1-*fj*TAL (KanR)	n/a	n/a	[1]
pACYCDuet-1 (CmR)	n/a	n/a	Addgene
pET21aDuet-1 (AmpR)	n/a	n/a	Addgene
pET28bDuet-1 (KanR)	n/a	n/a	Addgene
pET-pT7-seC3H-tT7 (KanR)	Gibson assembly	C3HvectorF:CGCCCGGACCTGATTAACCCGGGCACGTAActcgagtc tggtaaagaaaccgc<br>C3HvectorR:GTTCAGGCGACCAGCCGGTGCCGGAGAGGTAATC GTCATgccgctgctcggatcc	This Study
pACYC-pT7-atCOMT-tT7 (CmR)	Gibson Assembly	COMTvectorF:GTGTGAATCTGATTGAAC TCCTGAAGAAACTGTAActcgagtctggtaaag aaac<br>COMTvectorR:CGTCAGCTGCGTTTCGGC GGTCGAGCCCATggtatatctccttattaaagttaaac	This Study
pACYC-pT7-*ph*CFAT-pT7-*ph*EGS-tT7 (CmR)	Overlap pcr, Gibson Assembly	vectorF: CGTTGAGGAGTATCTGTCACATTTTGCCTAActcgagtctggtaaagaaaccgctg<br>vector: GTAACATGAAAATCGGTGTTGCCCATggtatatctccttattaaagttaaacaaaattatttctacaggg	This Study
pET-pT7-*sc*ADH6-rbs-*ll*CCR-pT7-*at*4CL1-tT7 (KanR)	n/a	n/a	This Study; Synthesized by Twist Bioscience
pET- pT7-*se*C3H-rbs-*at*COMT-tT7 (KanR)	Overlap pcr, Gibson Assembly	vector: CTGATTGAACTCCTGAAGAAACTGTAActcgagtctggtaaagaaaccg<br>vector: GAAGAGATAAATTGCACTGAAATCTAGAGTtcgtcgccgcacttatgac	This Study
pRSFDuet1-*fj*TAL (AmpR)	Gibson Assembly	Amplify pET21a F: CAAATATGTATCCGCTCATGAATTAATTCTTAttaccaatgcttaatcagtgagg<br>Amplify pET21a R: taagaattaattcatgagcggatacatatttg<br>Amplify pRSF-*fj*TAL F: CAAATATGTATCCGCTCATGAATTAATTCTTAttaccaatgcttaatcagtgagg<br>Amplify pRSF-*fj*TAL R: atgcttcaataatattgaaaaaggaagagt	This Study
pRSF-pT7-*fj*TAL-pT7-*se*C3H-rbs-*at*COMT-tT7 (AmpR)	Gibson Assembly	Amplify pT7-*se*C3H-rbs-*at*COMT-F: CATCTTAGTATATTAGTTAAGTATAAGAAGGAGATATACAATGACGATTACC TCTCCGG<br>Amplify pT7-*se*C3H-rbs-*at*COMT-R: GGTTTCTTTACCAGACTCGAGTTACAGTTTCTTCAGGAGTTCAATCAG<br>Amplify pRSF-fjTAL-F: CTGATTGAACTCCTGAAGAAACTGTAACTCGAGTCTGGTAAAGAAACC<br>Amplify pRSF-fjTAL-F: CCGGAGAGGTAATCGTCATTGTATATCTCCTTCTTATACTTAACTAATATACT AAGATG	This Study

Supplementary Table 2: Strains used in this study

Strain Name	Composition	Use	Source
BL21(DE3)	E. coli str. B F– ompT hsdS$_B$ (r$_B$–, m$_B$–) *gal dcm* (DE3)	Base Strain for Modules II and III	New England Biolabs
eBL01		Base Strain for Module I	[2]
SMB074	[eBL01]: pRSFDuet-*fj*TAL, pACYCDuet-1 (KanR, CmR)	Module I	This Study
SMB075	[BL21(DE3)]: pET-pT7-seC3H-tT7; pACYC-pT7-atCOMT-tT7 (KanR, CmR)	Module II, Eugenol production	This Study
SMB079	[BL21(DE3)]: pET-pT7-seC3H-tT7 (KanR)	Module II, Hydroxychavicol production	This Study
SMB146	[BL21(DE3)]: pACYC-pT7-*ph*CFAT-pT7-*ph*EGS-tT7 (CmR)	Conversion of Coniferyl Alcohol to Eugenol	This Study
SMB151	[BL21(DE3)]: pET-pT7-*sc*ADH6-rbs-*ll*CCR-pT7-*at*4CL1-tT7 (KanR)	Conversion of Ferulic Acid to Coniferyl Alcohol	This Study
SMB150	[BL21(DE3)]: pACYC-pT7-*ph*CFAT-pT7-*ph*EGS-tT7; pET-pT7-*sc*ADH6-rbs-*ll*CCR-pT7-*at*4CL1-tT7 (CmR, KanR)	Module III	This Study
SMB175	[BL21(DE3)]: pRSF-pT7-*fj*TAL-pT7-*se*C3H-rbs-*at*COMT-tT7; pACYC-pT7-*ph*CFAT-pT7-*ph*EGS-tT7; pET-pT7-*sc*ADH6-rbs-*ll*CCR-pT7-*at*4CL1-tT7 (AmpR, CmR, KanR)	Modules I-III in one strain	This Study

Sequences of genes used in this study (Purchased from Integrated DNA Technologies)

fjTAL (kindly provided as pRSFDuet-fjTAL from Dr. Kristala Prather):

ATGAACACCATCAACGAATATCTGAGCCTGGAAGAATTTGAAGCCATTATCTTTGGC
AATCAGAAAGTGACCATTAGTGATGTTGTTGTGAATCGCGTTAACGAGAGCTTTAAC
TTTCTGAAAGAATTTAGCGGCAACAAAGTGATCTATGGTGTGAATACCGGTTTTGGT
CCGATGGCACAGTATCGTATTAAAGAAAGCGATCAGATTCAGCTGCAGTATAATCTG
ATTCGTAGCCATAGCAGCGGCACCGGTAAACCGCTGAGTCCGGTTTGTGCAAAAGC
AGCAATTCTGGCACGTCTGAATACCCTGAGTCTGGGTAATAGCGGTGTTCATCCGAG
CGTTATTAATCTGATGAGCGAACTGATCAACAAAGATATTACACCGCTGATTTTTGA
ACATGGTGGTGTTGGTGCAAGCGGTGATCTGGTTCAGCTGAGCCATCTGGCACTGGT
TCTGATTGGTGAAGGTGAAGTTTTCTATAAAGGTGAACGTCGTCCGACACCGGAAGT
TTTTGAAATTGAAGGTCTGAAACCGATCCAGGTGGAAATTCGCGAAGGTCTGGCCCT
GATTAATGGCACCAGCGTTATGACCGGTATTGGTGTTGTTAATGTGTACCATGCAAA
AAAACTGCTGGATTGGAGCCTGAAAAGCAGCTGTGCAATTAATGAACTGGTTCAGG
CcTATGATGATCACTTTAGCGCAGAACTGAATCAGACCAAACGTCATAAAGGTCAGC
AAGAAATTGCACTGAAAATGCGTCAGAATCTGAGCGATAGCACCCTGATTCGCAAA
CGTGAAGATCATCTGTATAGCGGTGAAAACACCGAAGAAATCTTCAAAGAAAAAGT
GCAAGAGTATTATAGCCTGCGTTGTGTTCCGCAGATTCTGGGTCCGGTTCTGGAAAC
CATTAACAATGTTGCAAGCATTCTGGAAGATGAATTTAACAGCGCAAACGATAACC
CGATCATCGATGTTAAAAACCAGCATGTTTATCACGGTGGCAATTTTCATGGTGATT
ATATCAGCCTGGAAATGGATAAACTGAAAATCGTGATTACCAAACTGACCATGCTG
GCAGAACGTCAGCTGAATTATCTGCTGAATAGCAAAATTAACGAACTGCTGCCTCCG
TTTGTTAATCTGGGCACCCTGGGTTTTAACTTTGGTATGCAGGGTGTTCAGTTTACCG
CAACCAGCACCACCGCAGAAAGCCAGATGCTGAGCAATCCGATGTATGTTCATAGC

ATTCCGAACAATAATGATAACCAGGATATTGTTAGCATGGGCACCAATAGCGCAGTT
ATTACCAGCAAAGTTATCGAAAATGCCTTTGAAGTTCTGGCCATTGAAATGATTACC
ATTGTTCAGGCGATTGATTATCTGGGCCAGAAAGATAAAATCAGCAGCGTTAGCAA
AAAATGGTATGATGAAATCCGCAACATCATCCCGACCTTTAAAGAAGATCAGGTGA
TGTATCCGTTCGTGCAGAAAGTAAAAGACCACCTGATTAACAATtga

seC3H (codon optimized for expression in *E. coli* by Genscript):

ATGACGATTACCTCTCCGGCACCGGCTGGTCGCCTGAACAATGTCCGCCCGATGACG
GGTGAAGAATACCTGGAATCCCTGCGTGACGGCCGTGAAGTGTATATTTACGGTGA
ACGCGTCGATGACGTGACCACGCATCTGGCGTTCCGCAACAGCGTTCGTTCTATCGC
CCGCCTGTATGATGTCCTGCACGACCCGGCATCCGAAGGTGTTCTGCGTGTCCCGAC
CGATACGGGTAATGGTGGTTTTACCCATCCGTTTTTCAAAACGGCGCGTAGCTCTGA
AGACCTGGTGGCGGCCCGTGAAGCCATTGTCGGCTGGCAACGCCTGGTGTATGGCTG
GATGGGTCGTACCCCGGATTACAAAGCAGCGTTTTTCGGTACGCTGGACGCTAACGC
GGAATTTTATGGCCCGTTCGAAGCCAATGCACGTCGCTGGTATCGTGATGCACAGGA
ACGCGTTCTGTACTTCAACCATGCTATCGTTCACCCGCCGGTCGATCGTGACCGTCC
GGCTGATCGTACCGCAGACATTTGCGTCCATGTGGAAGAAGAAACGGATTCAGGCC
TGATCGTGTCGGGTGCCAAAGTGGTTGCAACCGGTTCTGCTATGACGAACGCGAATC
TGATTGCCCACTATGGTCTGCCGGTTCGCGATAAAAAATTTGGCCTGGTGTTCACCG
TTCCGATGAACAGTCCGGGTCTGAAACTGATCTGTCGTACCTCCTATGAACTGATGG
TGGCCACGCAGGGCTCACCGTTTGATTACCCGCTGAGTTCCCGCCTGGATGAAAATG
ACAGCATTATGATCTTTGATCGTGTTCTGGTCCCGTGGGAAAACGTTTTCATGTACG
ACGCAGGCGCGGCCAATAGCTTTGCTACCGGCTCTGGTTTCCTGGAACGCTTTACCT
TCCATGGTTGCACGCGTCTGGCAGTGAAACTGGATTTTATTGCAGGCTGTGTTATGA
AAGCTGTGGAAGTTACCGGCACCACGCACTTCCGCGGTGTTCAGGCGCAAGTCGGC
GAAGTGCTGAACTGGCGTGATGTCTTTTGGGGTCTGTCGGACGCTATGGCGAAAAGT
CCGAATTCCTGGGTGGGCGGTAGCGTTCAGCCGAACCTGAATTATGGCCTGGCCTAC
CGCACCTTTATGGGCGTGGGTTATCCGCGTATTAAAGAAATTATCCAGCAAACGCTG
GGCTCTGGTCTGATCTACCTGAACTCATCGGCAGCTGATTGGAAAAATCCGGACGTT
CGCCCGTATCTGGATCGTTACCTGCGCGGCAGTCGTGGTATTCAGGCAATCGATCGT
GTCAAACTGCTGAAACTGCTGTGGGACGCAGTGGGTACCGAATTCGCAGGTCGTCAT
GAACTGTATGAACGCAACTACGGCGGTGATCACGAAGGTATTCGTGTGCAGACCCT
GCAAGCCTATCAGGCAAATGGTCAAGCGGCCGCACTGAAAGGCTTTGCGGAACAGT
GTATGTCGGAATATGACCTGGATGGCTGGACCCGCCCGGACCTGATTAACCCGGGC
ACGtaa

atCOMT (codon optimized for expression in *E. coli* by Genscript):

ATGGGCTCGACCGCCGAAACGCAGCTGACGCCAGTGCAGGTTACCGATGACGAAGC
GGCACTGTTTGCCATGCAGCTGGCGTCCGCCTCGGTCTTGCCAATGGCGCTCAAAAG
CGCGTTGGAGCTGGATCTGCTCGAAATTATGGCGAAAAATGGCTCGCCGATGTCCCC
GACCGAGATCGCCAGTAAATTACCAACGAAAAATCCGGAGGCGCCGGTGATGCTCG
ACCGTATTCTGCGTCTTTTGACCTCGTATAGTGTTTTAACCTGCAGTAATCGTAAATT
ATCGGGCGACGGTGTGGAACGCATCTACGGTCTGGGCCCAGTTTGCAAATATCTGAC
GAAAAATGAGGACGGTGTTAGCATCGCGGCCCTGTGTCTGATGAACCAGGATAAAG

TTCTGATGGAATCCTGGTATCACCTCAAAGATGCGATCCTGGATGGCGGCATTCCGT
TTAACAAAGCCTATGGTATGAGCGCCTTCGAATATCATGGTACTGACCCACGTTTTA
ATAAAGTGTTTAACAATGGCATGAGTAATCATTCGACCATTACCATGAAAAAGATTT
TAGAGACTTACAAGGGCTTTGAAGGCTTAACCTCCCTGGTTGATGTGGGCGGCGGCA
TCGGCGCGACCCTGAAAATGATTGTGAGCAAATATCCGAATCTGAAAGGTATTAACT
TTGATCTTCCGCATGTTATCGAGGACGCACCGAGTCACCCAGGTATCGAGCATGTGG
GCGGCGACATGTTTGTGAGTGTTCCGAAGGGCGACGCGATCTTTATGAAATGGATTT
GTCATGATTGGAGTGATGAGCATTGTGTCAAATTTCTTAAAAATTGTTATGAATCGT
TACCAGAGGATGGTAAAGTTATTCTGGCAGAATGCATTTTACCAGAAACCCCGGATT
CCAGCTTGTCCACCAAACAGGTCGTGCACGTTGACTGTATCATGTTAGCCCATAACC
CAGGCGGCAAAGAGCGCACCGAAAAAGAATTCGAGGCGTTGGCGAAAGCAAGCGG
CTTCAAAGGTATCAAGGTTGTCTGTGATGCCTTTGGTGTGAATCTGATTGAACTCCTG
AAGAAACTGtaa

at4CL1 (codon optimized for expression in *E. coli* by Thermofisher GeneArt):

ATGGCACCCCAAGAACAAGCTGTATCACAGGTGATGGAAAAACAGAGCAACAACA
ATAACTCCGACGTCATTTTCCGCAGCAAGCTTCCAGACATCTACATCCCGAACCATC
TGTCATTACACGATTATATCTTCCAGAACATTAGCGAGTTCGCCACCAAACCGTGCC
TGATCAACGGCCCGACGGGTCACGTGTATACCTATTCCGATGTGCATGTTATCAGCC
GTCAGATCGCCGCGAACTTCCACAAGTTAGGTGTTAATCAAAACGATGTCGTTATGC
TGTTGCTTCCGAATTGTCCGGAATTCGTGCTATCTTTTCTGGCAGCGAGCTTTCGTGG
TGCGACCGCTACGGCTGCGAATCCGTTCTTCACCCCGGCAGAAATTGCCAAGCAGGC
AAAGGCTAGCAATACCAAGCTGATTATAACCGAGGCGCGTTATGTTGATAAAATAA
AGCCGCTGCAGAACGATGACGGCGTTGTGATTGTATGTATCGACGACAACGAAAGT
GTTCCGATTCCGGAAGGCTGCCTGCGTTTTACCGAACTCACACAGAGCACCACCGAG
GCTTCCGAAGTTATTGATAGCGTGGAAATTTCCCCGGACGACGTTGTGGCGCTGCCG
TATTCGTCTGGCACGACCGGCTTACCGAAAGGTGTCATGCTGACCCATAAAGGCTTG
GTTACCAGCGTCGCGCAACAAGTGGACGGCGAGAACCCGAATCTTTATTTTCACAGC
GATGACGTGATCTTATGCGTTCTGCCGATGTTTCACATTTACGCATTGAACTCGATTA
TGCTGTGCGGTCTGAGAGTGGGTGCGGCGATCCTGATTATGCCGAAATTTGAGATCA
ACCTGCTCCTGGAACTGATCCAGCGTTGTAAAGTGACCGTTGCTCCGATGGTTCCTC
CGATTGTGTTGGCCATCGCAAAATCTAGCGAAACCGAGAAGTACGACCTGAGCTCC
ATTCGTGTCGTTAAGTCCGGCGCGGCTCCGCTGGGTAAAGAGCTGGAGGATGCAGTT
AATGCCAAGTTCCCCAACGCCAAGCTGGGTCAAGGTTACGGTATGACGGAAGCGGG
TCCGGTCCTGGCAATGAGCCTGGGCTTTGCAAAAGAACCGTTTCCGGTGAAGTCTGG
CGCGTGCGGTACTGTGGTGCGTAATGCGGAGATGAAAATCGTGGACCCGGATACCG
GCGATTCCCTGAGTCGCAACCAGCCGGGTGAGATCTGCATCCGCGGTCATCAAATCA
TGAAAGGTTACCTAAACAATCCGGCGGCGACTGCGGAAACCATTGACAAGGACGGC
TGGCTGCATACCGGCGATATCGGTTTGATTGACGACGATGACGAATTGTTTATCGTC
GATCGTCTGAAAGAGCTGATCAAATACAAAGGATTCCAGGTTGCCCCAGCTGAGTT
GGAGGCGTTGTTGATCGGTCACCCTGACATCACCGATGTCGCGGTTGTTGCCATGAA
AGAGGAAGCTGCTGGTGAGGTGCCAGTGGCGTTTGTTGTTAAGTCAAAGGATAGCG
AGCTCTCTGAGGACGACGTGAAGCAATTCGTAAGCAAACAAGTGGTTTTCTACAAG
CGCATTAACAAAGTCTTCTTCACGGAAAGCATTCCGAAGGCGCCAAGCGGCAAGAT
CCTGCGCAAAGACCTGCGTGCAAAGCTGGCGAATGGCCTGtaa

llCCR (codon optimized for expression in *E. coli* by Thermofisher GeneArt):

ATGCCTGCAGCAGCACCGGCACCGACCGCAGCAAATACCACCAGTAGCGGTAGCGG
TCAGACCGTTTGTGTTACCGGTGCAGGCGGTTTTATTGCAAGCTGGATTGTTAAACT
GCTGCTGGAACGTGGTTATACCGTTCGTGGCACCGTTCGTAATCCGGATGATAGCAA
AAATAGCCATCTGAAAGAACTGGAAGGTGCAGAAGAACGTCTGACCCTGCATAAAG
TTGATCTGCTGGATCTGGAAAGCGTTAAAGCAGTTATTAATGGCTGTGATGGCATTA
TTCATACCGCAAGTCCGGTTACCGATAATCCGGAAGAAATGGTTGAACCGGCAGTTA
ATGGTGCCAAAAATGTTATTATTGCAGCAGCCGAAGCAAAAGTTCGTCGTGTTGTTT
TTACCAGCAGCATTGGTGCAGTTTATATGGATCCGAGCCGTAATATTGATGAAGTTG
TTGATGAAAGCTGTTGGAGCAATCTGGAATATTGCAAAAACACCAAAAACTGGTAC
TGCTATGGTAAAGCCGTTGCAGAACAGGCAGCATGGGATGAAGCCAAAGCACGTGG
TGTTGATCTGGTTGTTGTTAATCCGGTTCTGGTTCTGGGTCCTCTGCTGCAGAGCACC
ATGAATGCAAGCACCATTCATATTCTGAAATATCTGACCGGTAGCGCAAAAACCTAT
GCAAATGCAACCCAGGCATATGTGCATGTTAAAGATGTTGCACTGGCACATGTTCTG
GTTTATGAAATTCCGAGCGCAAGCGGTCGTTATCTGTGTAGCGAAAGCAGCCTGCAT
CGTGGTGAACTGGTTGAAATTCTGGCAAAATACTTTCCGGAATATCCGATTCCGACC
AAATGCTCCGATGAAAAAAATCCGCGTGCAAAAGCATATACCTTTAGCAATAAACG
CCTGAAAGATCTGGGCTTAGAATTTACACCGGTTCATCAGTGTCTGTATGACACCGT
TAAAAGCCTGCAGGATAAAGGTCATCTGCCGCTGCCGACAAAAtaa

scADH6 (codon optimized for expression in *E. coli* by Thermofisher GeneArt):

ATGAGCTACCCGGAAAAATTTGAAGGTATTGCAATTCAGAGCCACGAGGATTGGAA
AAATCCGAAAAAAACCAAATATGACCCGAAGCCGTTTTATGATCATGACATCGATA
TCAAAATTGAGGCCTGTGGTGTTTGTGGTAGCGATATTCATTGTGCAGCAGGTCATT
GGGGTAATATGAAAATGCCGCTGGTTGTTGGTCATGAAATTGTTGGTAAAGTTGTTA
AGCTGGGTCCGAAAAGCAATAGCGGTCTGAAAGTTGGTCAGCGTGTTGGTGTTGGT
GCACAGGTTTTTAGCTGTCTGGAATGTGATCGTTGCAAAAATGATAACGAACCGTAC
TGCACCAAATTCGTTACCACCTATAGCCAGCCGTATGAAGATGGTTATGTTAGCCAA
GGTGGTTATGCCAATTATGTTCGTGTGCATGAACATTTTGTTGTGCCGATTCCGGAA
AATATTCCGAGCCATCTGGCAGCACCGCTGCTGTGTGGTGGTCTGACCGTTTATAGT
CCGCTGGTTCGTAATGGTTGTGGTCCGGGTAAAAAAGTTGGTATTGTTGGTTTAGGT
GGTATTGGTAGCATGGGCACCCTGATTAGCAAAGCAATGGGTGCAGAAACCTATGT
TATTAGCCGTAGCAGCCGTAAACGTGAAGATGCAATGAAAATGGGTGCCGATCATT
ACATTGCAACCCTGGAAGAAGGTGATTGGGGAGAAAAATACTTTGATACCTTTGATC
TGATTGTTGTTTGTGCAAGCAGCCTGACCGATATCGATTTTAACATTATGCCGAAAG
CCATGAAAGTCGGTGGTCGTATTGTTAGCATTAGCATTCCGGAACAGCATGAAATGC
TGAGCCTGAAACCGTATGGCCTGAAAGCAGTGAGCATTAGCTATAGCGCACTGGGT
AGCATTAAAGAACTGAATCAGCTGCTGAAACTGGTGAGCGAAAAAGACATTAAAAT
CTGGGTTGAAACCCTGCCGGTTGGTGAAGCCGGTGTTCATGAAGCATTTGAACGTAT
GGAAAAAGGTGACGTGCGTTATCGTTTTACCCTGGTTGGTTATGATAAAGAGTTCAG
CGATtaa

phCFAT (codon optimized for expression in *E. coli* by Thermofisher GeneArt):

ATGGGCAACACCGATTTTCATGTTACCGTGAAAAAGAAAGAAGTTGTTGCAGCAGTT
CTGCCGATGCATCATGAACATTGGCTGCCGATGAGCAATCTGGATCTGCTGCTGCCT
CCGCTGGATTTTGGTGTGTTTTTTTGTTATAAACGCAGCAAAATCAACAACGACACC
AAAGATGATGACGAAACCATCAAAAAAGCACTGGCAGAAACCCTGGTTAGCTTTTA
TGCCCTGGCAGGCGAAGTTGTGTTTAATAGCCTGGGTGAACCGGAACTGCTGTGTAA
TAATCGTGGTGTGGATTTTTTTCATGCCTATGCCGATATTGAACTGAACAACCTGGAT
CTGTATCATCCTGATGTTAGCGTTCATGAAAAACTGATCCCGATCAAAAAACATGGT
GTTCTGAGCGTTCAGGTTACCGGTCTGAAATGTGGTGGTATTGTTGTTGGTTGTACCT
TTGATCATCGTGTTGCAGATGCATATAGCGCCAATATGTTTCTGGTTGCATGGGCAG
CAATTGCCCGTAAAGATAATAACATTAATACCGTGATTCCGAGCTTTCGTCGTAGCC
TGCTGAATCCGCGTCGTCCGCCTCAGTTTGATGATAGCTTTATTGATAGCACCTACGT
GTTTCTGAGCAGCCCTCCGAAACAGCCGAATGATGTTCTGACCAGCCGTGTGTATTA
TATCAACAGCCAAGAAATTAATCTGCTGCAGAGCCAGGCAACCCGTAATGGTAGCA
AACGTAGTAAACTGGAATGTTTTAGCGCCTTTCTGTGGAAAACCATTGCCGAAGGTG
GCATTGATGATTCAAAACGTTGCAAACTGGGTATTGTGGTTGATGGTCGTCAGCGTC
TGCGTCATGATAGCAGCACCACCATGAAAAATTACTTTGGTAATGTGCTGAGCGTGC
CGTATACCGAAGCAAGCGTTGGTCAGCTGAAACAGACACCGCTGGGTAAAGTTGCA
GATCTGGTTCATACCTGTCTGGATAATGTTGCCAATGAACATCACTTTCCGAGCCTG
ATTGATTGGGTTGAACTGCATCGTCCGCGTCAGGCAATTGTTAAAGTTTATTGTAAA
GACGAGTGCAACGATGAAGCAGCCATTGTTGTTAGCAGCGGTCTGCGTTTTCCGCTG
AGCCAGGTTAATTTTGGTTGGGGTTGTCCGGATTTTGGCAGCTATATCTTTCCGTGGG
GTGGTCAGACCGGCTATGTTATGCCGATGCCGAGTCCGAATAAAAACGGTGATTGG
ATTGTTTACATGCACCTTCAGAAAAAACATCTGGACCTGGTTGAAACCCGTGCACCG
CATATTTTTCATCCGCTGACCGCATGTTATCTGGATTTAACCGCAACCTATtaa

phEGS (codon optimized for expression in *E. coli* by Genscript):

ATGGCTGAAAAGTCAAAAATTCTAATAATAGGCGGTACAGGTTACATCGGTAAGTTT
GTGGTGGAGGCGAGCGCAAAGGCGGGCCATCCGACCTTCGTTCTGGTGCGTGAATC
TACCGTGAGCGATCCGGCTAAAGGCAAGATCGTGGAGTCGTTCAACAACAGCGGTG
TCACCATTCTCTACGGCGACCTGTACGACCACGAGTCCCTGGTTAAAGCGATTAAGC
AGGTTGACGTGGTTATTTCCACCGTCGGTCAGATGCAGCTGGCTGACCAGACGAAAA
TCATTGCCGCGATTAAAGAAGCGGGCAATATCAAGCGCTTTTTCCCGTCGGAATTTG
GTATGGATGTTGACAAAGTGAATGCGGTGGAACCGGCAAAAAGCACCTTCGCGATC
AAGGTCCAAATACGCCGTGCAATCGAGGCGGAAGGTATTCCGTATACTTATGTTAGC
AGCAATTGCTTTGCGGGTTACTTCCTGCCGACTTTGGTGCAACCGGGCGCAACCGAT
CCGCCACGTGATAAGGTGATCATTAGCGGGGATGGCAACGCAAAGGCGGTTTTTAA
CGAGGAGCACGATATCGGCACGTACACCATTAAGGCGGTAGACGACCCACGTACCT
TGAATAAGACCTTGTACATCAAGCCGCCTAAAAATACCCTGTCTTTCAACGAACTGG
TAGCCATCTGGGAAAAACTCATTGGCAAAACGCTGGAAAAAATCTATATCCCGGAA
GAGCAGATTCTGAAAGATATTGCGACGTCCCCGATTCCGATCAACATCATCCTTGCT
ATCAACCACAGTACCTTCGTTAAAGGTGACCAAACCAACTTTGTCATCGAGCCGAGC
TTCGGTGTTGAAGCTAGCGAGTTGTATCCGGATGTGAAGTACACCACCGTTGAGGAG
TATCTGTCACATTTTGCCtaa

EGS/CFAT overlap region (for use in overlap pcr to construct pACYC-pT7-*ph*CFAT-pT7-*ph*EGS-tT7 (CmR)):

GCATGTTATCTGGATTTAACCGCAACCTATTAAGAATTCTAATACGACTCACTATAG
GGGAATTGTGAGCGGATAACAATTCCCCTCTAGAAATAATTTTGTTTAACTTTAAGA
AGGAGATATACATATGGCTGAAAAGTCAAAAATTCTAATAATAGGCG

Additional Figures and Tables

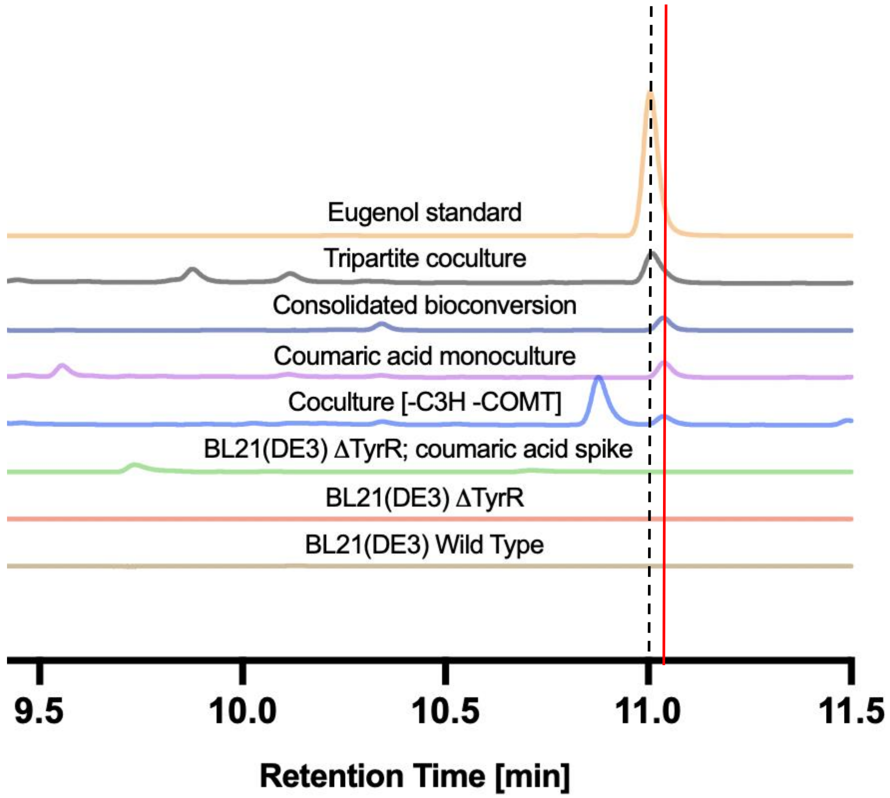

Supplementary Figure 1: Representative high-performance liquid chromatography (HPLC) traces from multiple bioproduction schemes. Eugenol retention time is represented by the dashed black line whereas the by-product produced by the consolidated bioconversion monoculture, coumaric acid monoculture, and coculture lacking C3H and COMT enzymes is represented by the solid red line.

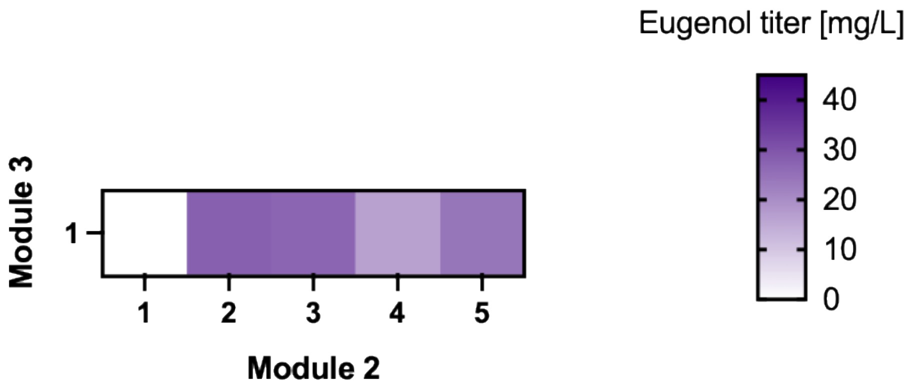

Supplementary Figure 2: Impact of additional alterations in Module II:III ratio on eugenol titer.
Heat map of eugenol titers achieved via tripartite coculture through expanded range of Module I: Module II ratios (Module I held at 1). Data are mean ± SD.; n=3 biological replicates. Source data are provided as source data file.

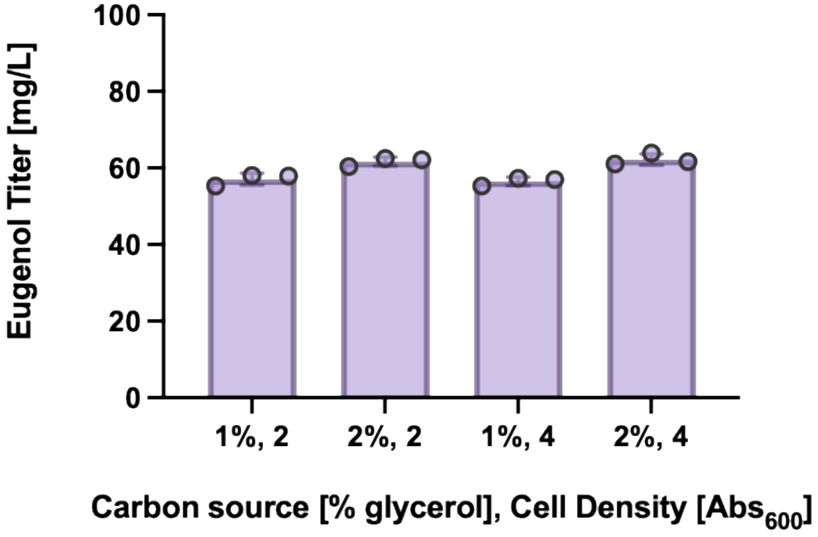

Supplementary Figure 3: Impact of altering carbon feed and cell density on eugenol titer.
Eugenol titer achieved for range of carbon source additions (percent weight per volume) as well as initial cell density of total consortia at start of IPTG induction. Data are mean ± SD.; n=3 biological replicates. Source data are provided as source data file.

		Ratio prior to IPTG induction	Ratio at start of production phase	Ratio at end of production phase
Consortia	*Strain*	*OD_{600} Basis*	*OD_{600} Basis*	*Colony PCR Count Basis*
**Ferulic Acid Co-culture**	Module I	1	1	1
	Module II	3	3.48	4
**Eugenol Tripartite Co-culture**	Module I	1	1	0
	Module II	3	3.48	6.75
	Module III	1	1.20	1

Supplementary Table 3: Quantification of strain ratios present at beginning and end of fermentation for ferulic acid and eugenol consortia. Ratios prior to induction were quantified using optical density at 600 nm (OD_{600}) measurements. Ratios at start of production phase (following 5 h of IPTG induction and immediately preceding mixing all strains together in production media) were quantified via OD_{600} measurements. Note that the pre-induction and pre-production ratios reflect both the relative cell densities of the strains before mixing as well as the use of 3x more starting culture for Module II relative to the other modules, as detailed in the methods section. Ratios at the end of 63 h fermentation were quantified via colony PCR after plating on selective agar. As all modules carried the same antibiotic resistance, ratios were determined from plates via colony PCR using primers specific to each module. 20 colonies and 31 colonies were screened for the ferulic acid and eugenol cocultures, respectively.

Coculture	mol_{Eug}/mol_{HC}	Relative titer HC
Tripartite	1.89 ± 0.29	1 ± 0.18
Tripartite [-COMT]	0	0.83 ± 0.28

Supplementary Table 4: Comparison of product profiles in tripartite coculture and tripartite coculture [-COMT]. Molar ratio of Eugenol/Hydroxychavicol after 46 h of culture between the two coculture formats is displayed in column 2. Relative titers of hydroxychavicol, using full tripartite coculture titer as the basis, is displayed in column 3. Eug = eugenol; HC = hydroxychavicol. Data are mean ± SD.; n=3 biological replicates.

References:

1. Haslinger, K. & Prather, K. L. J. Heterologous caffeic acid biosynthesis in *Escherichia coli* is affected by choice of tyrosine ammonia lyase and redox partners for bacterial Cytochrome P450. *Microbial Cell Factories* **19**, 26 (2020).
2. Johnston, T. G. *et al.* Compartmentalized microbes and co-cultures in hydrogels for on-demand bioproduction and preservation. *Nature Communications* **11**, 1–11 (2020).
