# Supplementary material for: A tripartite microbial co-culture system for de novo biosynthesis of diverse plant phenylpropanoids
Source: Nat Commun. 2023 Jul 24;14:4448. doi: 10.1038/s41467-023-40242-9 (PMC10366228; doi:10.1038/s41467-023-40242-9)
Supplement: Supplementary file 2 — Reporting Summary [file 41467_2023_40242_MOESM2_ESM.pdf]

Corresponding author(s): Alper, Hal S.

Last updated by author(s): Jul 2, 2023

Reporting Summary

Nature Portfolio wishes to improve the reproducibility of the work that we publish. This form provides structure for consistency and transparency in reporting. For further information on Nature Portfolio policies, see our Editorial Policies and the Editorial Policy Checklist.

Statistics

For all statistical analyses, confirm that the following items are present in the figure legend, table legend, main text, or Methods section.

n/a	Confirmed	
☐	☒	The exact sample size (*n*) for each experimental group/condition, given as a discrete number and unit of measurement
☐	☒	A statement on whether measurements were taken from distinct samples or whether the same sample was measured repeatedly
☒	☐	The statistical test(s) used AND whether they are one- or two-sided<br>*Only common tests should be described solely by name; describe more complex techniques in the Methods section.*
☒	☐	A description of all covariates tested
☐	☒	A description of any assumptions or corrections, such as tests of normality and adjustment for multiple comparisons
☐	☒	A full description of the statistical parameters including central tendency (e.g. means) or other basic estimates (e.g. regression coefficient) AND variation (e.g. standard deviation) or associated estimates of uncertainty (e.g. confidence intervals)
☒	☐	For null hypothesis testing, the test statistic (e.g. *F*, *t*, *r*) with confidence intervals, effect sizes, degrees of freedom and *P* value noted<br>*Give P values as exact values whenever suitable.*
☒	☐	For Bayesian analysis, information on the choice of priors and Markov chain Monte Carlo settings
☒	☐	For hierarchical and complex designs, identification of the appropriate level for tests and full reporting of outcomes
☒	☐	Estimates of effect sizes (e.g. Cohen's *d*, Pearson's *r*), indicating how they were calculated

Our web collection on statistics for biologists contains articles on many of the points above.

Software and code

Policy information about availability of computer code

Data collection	HPLC data were collected using the Chromeleon Chromatography Data System (CDS) Software (ThermoFisher). LC-MS data were collected using Agilent MassHunter Workstation LC/MS Data Acquisition for 6500 series Q-TOF (Version 10.1) .
Data analysis	HPLC data were analyzed using Microsoft Excel. LC-MS data were analyzed using Agilent MassHunter Workstation Qualitative Analysis (Version 10.0) software.

For manuscripts utilizing custom algorithms or software that are central to the research but not yet described in published literature, software must be made available to editors and reviewers. We strongly encourage code deposition in a community repository (e.g. GitHub). See the Nature Portfolio guidelines for submitting code & software for further information.

Data

Policy information about availability of data

All manuscripts must include a data availability statement. This statement should provide the following information, where applicable:

- Accession codes, unique identifiers, or web links for publicly available datasets
- A description of any restrictions on data availability
- For clinical datasets or third party data, please ensure that the statement adheres to our policy

A Data Availability Statement has been included in the manuscript which states "Data that support the findings of this study and the plasmids and strains used are available from the corresponding author upon request. Source data are provided with this paper."

Human research participants

Policy information about studies involving human research participants and Sex and Gender in Research.

Reporting on sex and gender	n/a
Population characteristics	n/a
Recruitment	n/a
Ethics oversight	n/a

Note that full information on the approval of the study protocol must also be provided in the manuscript.

Field-specific reporting

Please select the one below that is the best fit for your research. If you are not sure, read the appropriate sections before making your selection.

- [x] Life sciences
- [ ] Behavioural & social sciences
- [ ] Ecological, evolutionary & environmental sciences

For a reference copy of the document with all sections, see nature.com/documents/nr-reporting-summary-flat.pdf

Life sciences study design

All studies must disclose on these points even when the disclosure is negative.

Sample size	All fermentation experiments were conducted in biological triplicate as is the standard for high quality data in this field. The majority of other metabolic engineering publications by Nature journals (e.g. Srinivasan et al. Nature. 2020, Johnston et al. Nature Communications. 2020)
Data exclusions	No data were excluded from analysis
Replication	Experiments were replicated at least twice, with ability to obtain similar results confirmed for all published data. All attempts to replicate were successful.
Randomization	For each triplicate set of fermentation samples, separate colonies were used and chosen at random from an agar plate of the strain of interest. This ensured robustness in performance of engineered strains. Experimental groups were created out of individual strains and/or media conditions and thus were not chosen at random as the purpose of the study was to compare performance of different strains and media, as opposed to subjecting the same strain to different conditions.
Blinding	Blinding was not necessary as all experiments entailed output measurements of microbial fermentations in the form of HPLC or LC-MS traces which were not subject to interpretation. Given the absolute nature of the data, experimenters were not blinded. The subjects were not subject to blinding as they were bacterial strains, not humans.

Reporting for specific materials, systems and methods

We require information from authors about some types of materials, experimental systems and methods used in many studies. Here, indicate whether each material, system or method listed is relevant to your study. If you are not sure if a list item applies to your research, read the appropriate section before selecting a response.

Materials & experimental systems

n/a	Involved in the study	
x		Antibodies
x		Eukaryotic cell lines
x		Palaeontology and archaeology
x		Animals and other organisms
x		Clinical data
x		Dual use research of concern

Methods

n/a	Involved in the study	
x		ChIP-seq
x		Flow cytometry
x		MRI-based neuroimaging
